# Ten Steps to Conduct a Systematic Review

**DOI:** 10.7759/cureus.51422

**Published:** 2023-12-31

**Authors:** Ernesto Calderon Martinez, Jose R Flores Valdés, Jaqueline L Castillo, Jennifer V Castillo, Ronald M Blanco Montecino, Julio E Morin Jimenez, David Arriaga Escamilla, Edna Diarte

**Affiliations:** 1 Digital Health, Universidad Nacional Autónoma de México, Ciudad de Mexico, MEX; 2 General Medicine, Universidad Autonoma de Guadalajara, Guadalajara, MEX; 3 General Medicine, Universidad Autónoma de Guadalajara, Guadalajara, MEX; 4 Research, University of Texas Southwestern Medical Center, Dallas, USA; 5 General Medicine, Universidad Autónoma del Estado de México, Ciudad de Mexico, MEX; 6 Internal Medicine, Universidad Justo Sierra, Ciudad de Mexico, MEX; 7 Medicine, Universidad Autonoma de Sinaloa, Culiacan, MEX

**Keywords:** systematic review, guide, methodology, platforms, tools

## Abstract

This article introduces a concise 10-step guide tailored for researchers engaged in systematic reviews within the field of medicine and health, aligning with the imperative for evidence-based healthcare. The guide underscores the importance of integrating research evidence, clinical proficiency, and patient preferences. It emphasizes the need for precision in formulating research questions, utilizing tools such as PICO(S)(Population Intervention Comparator Outcome), PEO (Population Exposure Outcome), SPICE (setting, perspective, intervention/exposure/interest, comparison, and evaluation), and SPIDER (expectation, client group, location, impact, professionals, service and evaluation), and advocates for the validation of research ideas through preliminary investigations. The guide prioritizes transparency by recommending the documentation and registration of protocols on various platforms. It highlights the significance of a well-organized literature search, encouraging the involvement of experts to ensure a high-quality search strategy. The critical stages of screening titles and abstracts are navigated using different tools, each characterized by its specific advantages. This diverse approach aims to enhance the effectiveness of the systematic review process. In conclusion, this 10-step guide provides a practical framework for the rigorous conduct of systematic reviews in the domain of medicine and health. It addresses the unique challenges inherent in this field, emphasizing the values of transparency, precision, and ongoing efforts to improve primary research practices. The guide aims to contribute to the establishment of a robust evidence base, facilitating informed decision-making in healthcare.

## Introduction

The necessity of evidence-based healthcare, which prioritizes the integration of top-tier research evidence, clinical proficiency, and patient preferences, is increasingly recognized [1,2]. Due to the extensive amount and varied approaches of primary research, secondary research, particularly systematic reviews, is required to consolidate and interpret this information with minimal bias [3,4]. Systematic reviews, structured to reduce bias in the selection, examination, and consolidation of pertinent research studies, are highly regarded in the research evidence hierarchy. The aim is to enable objective, repeatable, and transparent healthcare decisions by reducing systematic errors.

To guarantee the quality and openness of systematic reviews, protocols are formulated, registered, and published prior to the commencement of the review process. Platforms such as PROSPERO (International Prospective Register of Systematic Reviews) aid in the registration of systematic review protocols, thereby enhancing transparency in the review process [5]. High-standard reviews comply with stringent peer review norms, ensuring that methodologies are revealed beforehand, thus reducing post hoc alterations for objective, repeatable, and transparent outcomes [6].

Nonetheless, the practical execution of systematic reviews, particularly in the field of medicine and health, poses difficulties for researchers. To address this, a succinct 10-step guide is offered to both seasoned and novice researchers, with the goal of improving the rigor and transparency of systematic reviews.

## Technical report

Step 1: structure of your topic

When developing a research question for a systematic review or meta-analysis (SR/MA), it is essential to precisely outline the objectives of the study, taking into account potential effect modifiers. The research question should concentrate on and concisely explain the scientific elements and encapsulate the aim of the project.

Instruments such as PICO(S)(Population Intervention Comparator Outcome), PEO (Population Exposure Outcome), SPICE (setting, perspective, intervention/exposure/interest, comparison, and evaluation), and SPIDER (expectation, client group, location, impact, professionals, service and evaluation) assist in structuring research questions for evidence-based clinical practice, qualitative research, and mixed-methods research [7-9]. A joint strategy of employing SPIDER and PICO is suggested for exhaustive searches, subject to time and resource constraints. PICO and SPIDER are the frequently utilized tools. The selection between them is contingent on the research’s nature. The ability to frame and address research questions is crucial in evidence-based medicine. The "PICO format" extends to the "PICOTS" (Population Intervention Comparator Outcome Time Setting) (Table 1) design. Explicit delineation of these components is critical for systematic reviews, ensuring a balanced and pertinent research question with broad applicability.

**Table 1:** PICOTS format This table gives a breakdown of the mnemonic for the elements required to formulate an adequate research question. Utilizing this mnemonic leads to a proper and non-biased search. Examples extracted from “The use and efficacy of oral phenylephrine versus placebo on adults treating nasal congestion over the years in a systematic review” [10]. RCT, randomized control trial; PICOTS, Population Intervention Comparator Outcome Time Setting

Structure	Meaning	Example	Inclusion criteria	Exclusion criteria
P	P (Population and/or Patient and/or Problem): It refers to the people in/for whom the systematic review is expected to be applied.	Adults’ population >18 years and <65 years	Adults between 18 and 65 years	Elderly, pediatrics, pregnant
I	I (Intervention): In the context of systematic reviews examining the effects of treatment. In other words, it encompasses medicines, procedures, health education, public health measures, or bundles/combinations. It also includes preventive measures like vaccination, prophylaxis, health education tools, and packages of such interventions. In some cases, intervention is not something that the investigators administer, and the investigators are merely observing the effects. Therefore, (I) can be better expressed as ‘Exposure’ abbreviated as (E). Diagnostic tests, prognostic markers, and condition prevalence can represent exposure.	Administration of oral phenylephrine	Oral administration of phenylephrine [10]	IV administration of phenylephrine, nasal phenylephrine
C	C (Comparison): It refers to the comparison of two groups; it can be people not receiving the intervention and those receiving an alternate intervention, placebo, or nothing. However, for some study designs and/or research questions, including a comparison may not be feasible.	Placebo, standard care, or no treatment	Phenylephrine vs. placebo	Phenylephrine in combination with another medication. Phenylephrine in comparison with other medication
O	O (Outcome): This refers to the effect intervention (I) has on the selected population (P) in comparison to the comparison (C). Most systematic reviews focus on efficacy, safety, and sometimes cost. When a systematic review focuses on diagnostic tests, the aim is to identify accuracy, reliability, and cost.	Symptoms like nasal congestion and nasal airway resistance	Nasal congestion management	Other allergy-related symptoms
T	T (Time Frame): The outcomes are only relevant when it is evaluated in a specific time frame.	Over the years	Taking medication over some time	One day, one week
S	S (Study Design): A study design is a specific protocol that allows the conduction of the study, allowing the investigator to translate the conceptual hypothesis research question into an operational one.	RCTs	RCT	Letters to the editor, case-control trials, observational

While there are various formats like SPICE and ECLIPSE, PICO continues to be favored due to its adaptability across research designs. The research question should be stated in the introduction of a systematic review, laying the groundwork for impartial interpretations. The PICOTS template is applicable to systematic reviews that tackle a variety of research questions.

Validation of the Idea

To bolster the solidity of our research, we advocate for the execution of preliminary investigations and the validation of ideas. An initial exploration, especially in esteemed databases like PubMed, is vital. This process serves several functions, including the discovery of pertinent articles, the verification of the suggested concept, the prevention of revisiting previously explored queries, and the assurance of a sufficient collection of articles for review.

Moreover, it is crucial to concentrate on topics that tackle significant healthcare challenges, align with worldwide necessities and principles, mirror the present scientific comprehension, and comply with established review methodologies. Gaining a profound comprehension of the research field through pertinent videos and discussions is crucial for enhancing result retrieval. Overlooking this step could lead to the unfortunate unearthing of a similar study published earlier, potentially leading to the termination of our research, a scenario where precious time would be squandered on an issue already thoroughly investigated.

For example, during our initial exploration using the terms “Silymarin AND Liver Enzyme Levels” on PubMed, we discovered a systematic review and meta-analysis discussing the impact of Silymarin on liver enzyme levels in humans [11]. This discovery acts as a safety net because we will not pursue this identical idea/approach and face rejection; instead, we can rephrase a more sophisticated research question or objective, shifting the focus on evaluating different aspects of the same idea by just altering a part of the PICOTS structure. We can evaluate a different population, a different comparator, and a different outcome and arrive at a completely novel idea. This strategic method guarantees the relevance and uniqueness of our research within the scientific community.

Step 2: databases

This procedure is consistently executed concurrently. A well-orchestrated and orderly team is essential for primary tasks such as literature review, screening, and risk of bias evaluation by independent reviewers. During the study inclusion phase, if disagreements arise, the involvement of a third independent reviewer often becomes vital for resolution. The team’s composition should strive to include individuals with a variety of skills.

The intricacy of the research question and the expected number of references dictate the team’s size. The final team structure is decided after the definitive search, with the participation of independent reviewers dependent on the number of hits obtained. It is crucial to maintain a balance of expertise among team members to avoid undue influence from a specific group of experts. Importantly, a team requires a competent leader who may not necessarily be the most senior member or a professor. The leader plays a central role in coordinating the project, ensuring compliance with the study protocol, keeping all team members updated, and promoting their active involvement.

**Figure 1:**
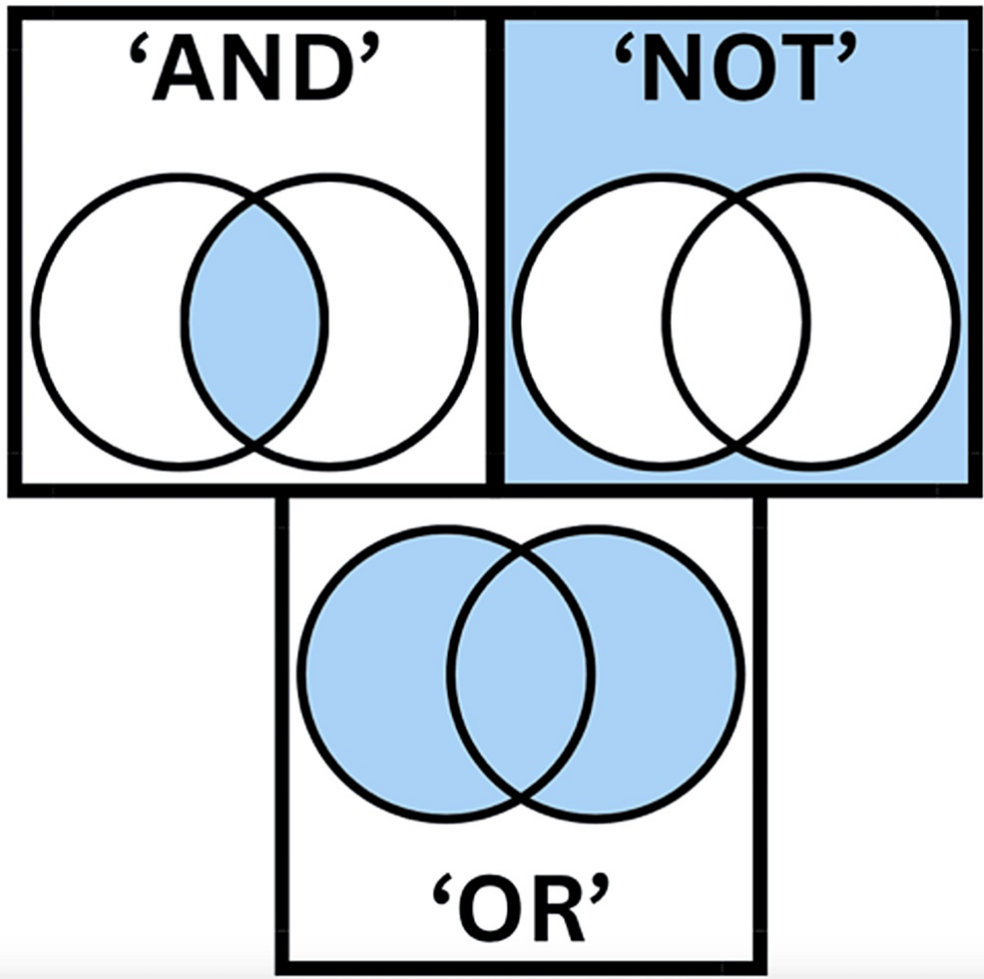
Boolean operators Boolean operators help break down and narrow down the search. "AND" will narrow your search so you get fewer results. It tells the database that your search results must include every one of your search terms. "OR" means MORE results. OR tells the database that you want results that mention one or both of your search terms. "NOT" means you are telling the database that you wish to have information related to the first term but not the second. Image credits to authors of the articles (Created on www.canva.com)

Establishing solid selection criteria is the foundational step in a systematic review. These criteria act as the guiding principles during the screening process, ensuring a focused approach that conserves time, reduces errors, and maintains transparency and reproducibility, being a primary component of all systematic review protocols. Carefully designed to align with the research question, as in Table 1, the selection criteria cover a range of study characteristics, including design, publication date, and geographical location. Importantly, they incorporate details related to the study population, exposure and outcome measures, and methodological approaches. Concurrently, researchers must develop a comprehensive search strategy to retrieve eligible studies. A well-organized strategy using various terms and Boolean operators is typically required (Figure 1). It involves crafting specific search queries for different online databases, such as Embase, MEDLINE, Web of Science, and Google Scholar. In these searches, we can include singulars and plurals of the terms, misspellings of the terms, and related terms, among others. However, it is crucial to strike a balance, avoiding overly extensive searches that yield unnecessary results and insufficient searches that may miss relevant evidence. In this process, collaborating with a librarian or search specialist improves the quality and reproducibility of the search. For this, it is important to understand the basic characteristics of the main databases (Table 2). It is important for the team to include in their methodology how they will collect the data and the tools they will use for their entire protocol so that there is a consensus about this among all of them.

**Table 2:** Databases' main characteristics Principal databases where the main articles of the whole body of the research can be gathered. This is an example of specialities and it can be used for the researchers to have a variety of databases to work. NLM, National Library of Medicine; ICTRP, International Clinical Trials Registry Platform; LILACS, Literatura Latino-Americana e do Caribe em Ciências da Saúde

Database	Principal characteristics
PubMed [12,13]	A free search engine accessing primarily the MEDLINE database of references and abstracts on life sciences and biomedical topics. It is maintained by the United States NLM at the National Institutes of Health
EMBASE [14]	A biomedical and pharmacological database containing bibliographic records with citations, abstracts, and indexing derived from biomedical articles in peer-reviewed journals. It is especially strong in its coverage of drug and pharmaceutical research.
Cochrane [15]	A database of systematic reviews. It includes reliable evidence from Cochrane and other systematic reviews of clinical trials
Google Scholar [16]	A freely accessible web search engine that indexes the full text or metadata of scholarly literature across an array of publishing formats and disciplines.
Web of Science [17]	A research database used for citation analysis. It provides access to multiple databases including the Science Citation Index, the Social Sciences Citation Index, and the Arts and Humanities Citation Index.
Science Direct [18]	A full-text scientific database offering journal articles and book chapters from more than 2,500 peer-reviewed journals and more than 11,000 books.
PsychINFO [15]	An electronic bibliographic database providing abstracts and citations to the scholarly literature in the psychological, social, behavioral, and health sciences.
ICTRP [19]	ICTRP is a database of clinical trials being conducted around the world. It is maintained by the World Health Organization.
Clinical Trials [20]	A database of privately and publicly funded clinical studies conducted around the world. It is provided by the United States NLM.
LILACS [21]	The LILACS is an online bibliographic database of scientific and medical publications maintained by the Latin American and Caribbean Center on Health Sciences Information.

Documenting and registering the protocol early in the research process is crucial for transparency and avoiding duplication. The protocol serves as recorded guidance, encompassing elements like the research question, eligibility criteria, intervention details, quality assessment, and the analysis plan. Before uploading to registry sites, such as PROSPERO, it is advisable to have the protocol reviewed by the principal investigator. The comprehensive study protocol outlines research objectives, design, inclusion/exclusion criteria, electronic search strategy, and analysis plan, providing a framework for reviewers during the screening process. These are steps previously established in our process. Registration can be done on platforms like PROSPERO 5 for health and social care reviews or Cochrane 3 for interventions.

Step 3: search

In the process of conducting a systematic review, a well-organized literature search is a pivotal step. It is suggested to incorporate at least two to four online databases, such as Embase, MEDLINE, Web of Science, and Cochrane. As mentioned earlier, formulating search strategies for each database is crucial due to their distinct requirements. In line with AMSTAR (A Measurement Tool to Assess Systematic Reviews) guidelines, a minimum of two databases should be explored in systematic reviews/meta-analyses (SR/MA), but increasing this number improves the accuracy of the results [22]. We advise including databases from China as most studies exclude databases from this demographic [9]. The choice of databases, like Cochrane or ICTRP, is dependent on the review questions, especially in the case of clinical trials. These databases cater to various health-related aspects, and researchers should select based on the research subject. Additionally, it is important to consider unique search methods for each database, as some may not support the use of Boolean operators or quotations. Detailed search strategies for each database, including customization based on specific attributes, are provided for guidance. In general, systematic reviews involve searching through multiple databases and exploring additional sources, such as reference lists, clinical trial registries, and databases of non-indexed journals, to ensure a comprehensive review of both published and, in some instances, unpublished literature.

It is important to note that the extraction of information will also vary among databases. However, our goal is to obtain a RIS, BibText, CSV, bib, or txt file to import into any of the tools we will use in subsequent steps.

Step 4: tools

It is necessary to upload all our reference files into a predetermined tool like Rayyan, Covidence, EPPI, CADIMA, and DistillerSR for the collection and management of records (Table 3). The subsequent step entails the elimination of duplicates using a particular method. Duplicates are recognized if they have the same title and author published in the same year or if they have the same title and author published in the same journal. Tools such as Rayyan or Covidence assist in automatically identifying duplicates. The eradication of duplicate records is vital for lessening the workload during the screening of titles and abstracts.

**Table 3:** Tools for title, abstract, and full-text screening The tools described above use artificial intelligence to help create keywords according to the inclusion and exclusion criteria defined previously by the researcher. This tool will help to reduce the amount of time to rule in or out efficiently.

Tool	Description	Key Features	Usage	Cost	Duplicate removal	Article screening	Critical appraisal	Assist with reporting
Covidence [23]	Web-based software for managing systematic review projects.	Streamlined screening and data extraction processes; collaboration features for team members; integration with reference management tools; real-time project tracking.	Systematic reviews and evidence synthesis projects.	Subscription-based, pricing varies.	Yes	Yes	Yes	Yes
Rayyan [24]	A web application designed for systematic review screening and study selection.	User-friendly interface for importing, screening, and organizing studies; collaboration tools for multiple reviewers; supports a variety of file formats.	Screening and study selection in systematic reviews.	Free with limitations; Premium plans available.	No	Yes	No	Limited
EPPI-Reviewer [25]	Software for managing the review process, with a focus on systematic reviews and other forms of evidence synthesis.	Comprehensive data extraction and synthesis capabilities; customizable review processes; integration with reference management tools.	Systematic reviews, evidence synthesis, and meta-analysis.	Subscription-based, pricing varies.	Yes	Yes	Yes	Yes
CADIMA [26]	A web-based systematic review software platform.	Customizable review workflow; collaboration tools for team members; integrated data extraction and synthesis features; real-time project tracking.	Systematic reviews and evidence synthesis projects.	Subscription-based, pricing varies.	Yes	Yes	Yes	Limited
DistillerSR [27]	Online systematic review software for data extraction and synthesis.	Streamlined data extraction and synthesis tools; collaboration features for team members; real-time progress tracking; integration with reference management tools.	Systematic reviews and evidence synthesis projects.	Subscription-based, pricing varies.	Yes	Yes	Yes	Yes

Step 5: title and abstract screening

The process of a systematic review encompasses several steps, which include screening titles and abstracts and applying selection criteria. During the phase of title and abstract screening, a minimum of two reviewers independently evaluate the pertinence of each reference. Tools like Rayyan, Covidence, and DistillerSR are suggested for this phase due to their effectiveness. The decisions to further assess retrieved articles are made based on the selection criteria. It is recommended to involve at least three reviewers to minimize the likelihood of errors and resolve disagreements.

In the following stages of the systematic review process, the focus is on acquiring full-text articles. Numerous search engines provide links for free access to full-text articles, and in situations where this is not feasible, alternative routes such as ResearchGate are pursued for direct requests from authors. Additionally, a manual search is carried out to decrease bias, using methods like searching references from included studies, reaching out to authors and experts, and exploring related articles in PubMed and Google Scholar. This manual search is vital for identifying reports that might have been initially overlooked. The approach involves independent reviewing by assigning specific methods to each team member, with the results gathered for comparison, discussion, and minimizing bias.

Step 6: full-text screening

The second phase in the screening process is full-text screening. This involves a thorough examination of the study reports that were selected after the title and abstract screening stage. To prevent bias, it is essential that three individuals participate in the full-text screening. Two individuals will scrutinize the entire text to ensure that the initial research question is being addressed and that none of the previously determined exclusion criteria are present in the articles. They have the option to "include" or "exclude" an article. If an article is "excluded," the reviewer must provide a justification for its exclusion. The third reviewer is responsible for resolving any disagreements, which could arise if one reviewer "excludes" an article that another reviewer "includes." The articles that are "included" will be used in the systematic review.

The process of seeking additional references following the full-text screening in a systematic review involves identifying other potentially relevant studies that were not found in the initial literature search. This can be achieved by reviewing the reference lists of the studies that were included after the full-text screening. This step is crucial as it can help uncover additional studies that are relevant to your research question but might have been overlooked in the initial database search due to variations in keywords, indexing terms, or other factors [15]. 

A PRISMA (Preferred Reporting Items for Systematic Reviews and Meta-Analyses) chart, also referred to as a PRISMA flow diagram, is a visual tool that illustrates the steps involved in an SR/MA. These steps encompass the identification, screening, evaluation of eligibility, and inclusion of studies.

The PRISMA diagram provides a detailed overview of the information flow during the various stages of an SR/MA. It displays the count of records that were identified, included, and excluded, along with the reasons for any exclusions.

The typical stages represented on a PRISMA chart are as follows: 1) identification: this is where records are discovered through database searches. 2) screening: this stage involves going through the records after removing any duplicates. 3) eligibility: at this stage, full-text articles are evaluated for their suitability. 4) included: this refers to the studies that are incorporated into the qualitative and quantitative synthesis. The PRISMA chart serves as a valuable tool for researchers and readers alike, aiding in understanding the process of study selection in the review and the reasons for the exclusion of certain studies. It is usually the initial figure presented in the results section of your systematic review [4].

Step 7: data extraction

As the systematic review advances, the subsequent crucial steps involve data extraction from the studies included. This process involves a structured data extraction from the full texts included, guided by a pilot-tested Excel sheet, which aids two independent reviewers in meticulously extracting detailed information from each article [28]. This thorough process offers an initial comprehension of the common characteristics within the evidence body and sets the foundation for the following analytical and interpretive synthesis. The participation of two to three independent reviewers ensures a holistic approach, including the extraction of both adjusted and non-adjusted data to account for potential confounding factors in future analyses. Moreover, numerical data extracted, such as dichotomous or continuous data in intervention reviews or information on true and false results in diagnostic test reviews, undergoes a thorough process. The extracted data might be suitable for pooled analysis, depending on sufficiency and compatibility. Difficulties in harmonizing data formats might occur, and systematic review authors might resort to communication with study authors to resolve these issues and enhance the robustness of the synthesis. This multi-dimensional data extraction process ensures a comprehensive and nuanced understanding of the included studies, paving the way for the subsequent analysis and synthesis phases.

Step 8: risk of bias assessment

To conduct a risk of bias in medical research, it is crucial to adhere to a specific sequence: choose tools that are specifically designed for systematic reviews. These tools should have proven acceptable validity and reliability, specifically address items related to methodological quality (internal validity), and ideally be based on empirical evidence of bias [29]. These tools should be chosen once the full text is obtained. For easy organization, it can be helpful to compile a list of the retrieved articles and view the type of study because it is necessary to understand how to select and organize each one. The most common tools to evaluate the risk of bias can be found in Table 4.

**Table 4:** Tools to assess risk of bias The table summarizes some of the different tools to appraise the different types of studies and their main characteristics. ROB, risk of bias; RRB, risk of reporting bias; AMSTAR; A Measurement Tool to Assess Systematic Reviews; GRADE, Grading of Recommendations Assessment, Development and Evaluations; ROBINS, risk of bias in non-randomized studies; RCT, randomized controlled trials

Tool	Description of the appraisal studies
Cochrane RoB2 Tool [31]	Widely used in both Cochrane and other systematic reviews. It replaces the notion of assessing study quality with that of assessing the risk of bias (RoB) 2 tool, considers biases arising at different stages of a trial (randomization process, deviation from intended intervention, missing outcome data, measurement of the outcome, and selection of the report result). It assesses RCT individually and in clusters. it also asses crossover RCT and cluster RCT
AHQR RRB [22]	Evaluates the risk of reporting bias and outcome reporting bias in a systematic review
AMSTAR 2 [32]	Assess the methodological quality of systematic reviews Including both randomized and non-randomized studies of healthcare interventions. Useful in the context of real-world observational evidence
Newcastle-Ottawa Quality Assessment Scale case-control studies [33]	Evaluate case-control studies. Assess the quality of non-randomized studies. Useful in the evaluation of the methodological quality of case-control studies. It provides a semi-quantitative measure of study quality that can be used to inform the interpretation of findings in a systematic review
GRADE [34]	It is used to assess the quality of evidence and the strength of recommendations in healthcare
ROBINS [35]	Tool used to assess the risk of bias in non-randomized studies. Two types of this tool (ROBINS-I and ROBINS-E). ROBINS-I assesses the risk of bias in the results of non-randomized studies that compare the health effects of two or more interventions; it evaluates the estimates of the effectiveness or safety (benefit or harm) of an intervention from studies that did not use randomization to allocate interventions. ROBINS-E provides a structured approach to assess the risk of bias in observational epidemiological studies, designed primarily for use in the context of a systematic review. Evaluates the effects of exposure (including environmental, occupational, and behavioral exposures) on human health. Both tools share many characteristics with the RoB2 tool. They are structured into a fixed set of domains of bias (signaling questions that inform the risk of bias judgments and overall risk of bias judgments). The seven domains of bias addressed are confounding, selection of participants, classification of intervention, deviations from intended interventions, missing data, measurement of outcomes, and selection of reported results. After completing all seven bias domains, an overall judgment is made for each three of the above-mentioned considerations.

After choosing the suitable tool for the type of study, you should know that a good risk of bias should be transparent and easily replicable. This necessitates the review protocol to include clear definitions of the biases that will be evaluated [30].

The subsequent step in determining the risk of bias is to understand the different categories of risk of bias. This will explicitly assess the risk of selection, performance, attrition, detection, and selective outcome reporting biases. It allows for separate risk of bias ratings by the outcome to account for the outcome-specific variations in detection bias and specific outcome reporting bias.

Keep in mind that assessing the risk of bias based on study design and conduct rather than reporting is very important. Poorly reported studies may be judged as unclear risk of bias. Avoid presenting the risk of bias assessment as a composite score. Finally, classifying the risk of bias as "low," "medium," or "high" is a more practical way to proceed. Methods for determining an overall categorization for the study limitations should be established a priori and documented clearly.

As a concluding statement or as a way to summarize the risk of bias, the assessment is to evaluate the internal validity of the studies included in the systematic review. This process helps to ensure that the conclusions drawn from the review are based on high-quality, reliable evidence.

Step 9: synthesis

This step can be broken down to simplify the concept of conducting a descriptive synthesis of a systematic review. 1) inclusion of studies: the final count of primary studies included in the review is established based on the screening process. 2) flowchart: the systematic review process flow is summarized in a flowchart. This includes the number of references discovered, the number of abstracts and full texts screened, and the final count of primary studies included. 3) study description: the characteristics of the included studies are detailed in a table in the main body of the manuscript. This includes the populations studied, types of exposures, intervention details, and outcomes. 4) results: if a meta-analysis is not possible, the results of the included studies are described. This includes the direction and magnitude of the effect, consistency of the effect across studies, and the strength of evidence for the effect. 5) reporting bias check: reporting bias is a systematic error that can influence the results of a systematic review. It happens when the nature and direction of the results affect the dissemination of research findings. Checking for this bias is an important part of the review process. 6) result verification: the results of the included studies should be verified for accuracy and consistency [36,37]. The descriptive synthesis primarily relies on words and text to summarize and explain the findings, necessitating careful planning and meticulous execution. 

Step 10: manuscript

When working on a systematic review and meta-analysis for submission, it is essential to keep the bibliographic database search current if more than six to 12 months have passed since the initial search to capture newly published articles. Guidelines like PRISMA and MOOSE provide flowcharts that visually depict the reporting process for systematic reviews and meta-analyses, promoting transparency, reproducibility, and comparability across studies [4,38]. The submission process requires a comprehensive PRISMA or MOOSE report with these flowcharts. Moreover, consulting with subject matter experts can improve the manuscript, and their contributions should be recognized in the final publication. A last review of the results' interpretation is suggested to further enhance the quality of the publication.

The composition process is organized into four main scientific sections: introduction, methods, results, and discussion, typically ending with a concluding section. After the manuscript, characteristics table, and PRISMA flow diagram are finalized, the team should forward the work to the principal investigator (PI) for comprehensive review and feedback. Finally, choosing an appropriate journal for the manuscript is vital, taking into account factors like impact factor and relevance to the discipline. Adherence to the author guidelines of journals is crucial before submitting the manuscript for publication.

## Discussion

The report emphasizes the increasing recognition of evidence-based healthcare, underscoring the integration of research evidence. The acknowledgment of the necessity for systematic reviews to consolidate and interpret extensive primary research aligns with the current emphasis on minimizing bias in evidence synthesis. The report highlights the role of systematic reviews in reducing systematic errors and enabling objective and transparent healthcare decisions. The detailed 10-step guide for conducting systematic reviews provides valuable insights for both experienced and novice researchers. The report emphasizes the importance of formulating precise research questions and suggests the use of tools for structuring questions in evidence-based clinical practice.

The validation of ideas through preliminary investigations is underscored, demonstrating a thorough approach to prevent redundancy in research efforts. The report provides a practical example of how an initial exploration of PubMed helped identify an existing systematic review, highlighting the importance of avoiding duplication. The systematic and well-coordinated team approach in the establishment of selection criteria, development of search strategies, and an organized methodology is evident. The detailed discussion on each step, such as data extraction, risk of bias assessment, and the importance of a descriptive synthesis, reflects a commitment to methodological rigor.

## Conclusions

The systematic review process is a rigorous and methodical approach to synthesizing and evaluating existing research on a specific topic. The 10 steps we followed, from defining the research question to interpreting the results, ensured a comprehensive and unbiased review of the available literature. This process allowed us to identify key findings, recognize gaps in the current knowledge, and suggest areas for future research. Our work contributes to the evidence base in our field and can guide clinical decision-making and policy development. However, it is important to remember that systematic reviews are dependent on the quality of the original studies. Therefore, continual efforts to improve the design, reporting, and transparency of primary research are crucial.
